# Elucidating variations in the nucleotide sequence of Ebola virus associated with increasing pathogenicity

**DOI:** 10.1186/s13059-014-0540-x

**Published:** 2014-11-22

**Authors:** Stuart D Dowall, David A Matthews, Isabel García-Dorival, Irene Taylor, John Kenny, Christiane Hertz-Fowler, Neil Hall, Kara Corbin-Lickfett, Cyril Empig, Kyle Schlunegger, John N Barr, Miles W Carroll, Roger Hewson, Julian A Hiscox

**Affiliations:** Public Health England, Porton Down, Salisbury, SP4 0JG UK; School of Molecular and Cellular Medicine, University of Bristol, Bristol, BS8 1TD UK; Institute of Infection and Global Health, University of Liverpool, Liverpool, L3 5RF UK; Institute of Integrative Biology, University of Liverpool, Liverpool, L69 7ZB UK; Peregrine Pharmaceuticals, Inc, Tustin, CA 92780 USA; School of Molecular and Cellular Biology, University of Leeds, Leeds, LS2 9JT UK; Honorary Professor in Vaccinology, University of Southampton, Southampton, UK

## Abstract

**Background:**

Ebolaviruses cause a severe and often fatal haemorrhagic fever in humans, with some species such as Ebola virus having case fatality rates approaching 90%. Currently, the worst Ebola virus outbreak since the disease was discovered is occurring in West Africa. Although thought to be a zoonotic infection, a concern is that with increasing numbers of humans being infected, Ebola virus variants could be selected which are better adapted for human-to-human transmission.

**Results:**

To investigate whether genetic changes in Ebola virus become established in response to adaptation in a different host, a guinea pig model of infection was used. In this experimental system, guinea pigs were infected with Ebola virus (EBOV), which initially did not cause disease. To simulate transmission to uninfected individuals, the virus was serially passaged five times in naïve animals. As the virus was passaged, virulence increased and clinical effects were observed in the guinea pig. An RNAseq and consensus mapping approach was then used to evaluate potential nucleotide changes in the Ebola virus genome at each passage.

**Conclusions:**

Upon passage in the guinea pig model, EBOV become more virulent, RNA editing and also coding changes in key proteins become established. The data suggest that the initial evolutionary trajectory of EBOV in a new host can lead to a gain in virulence. Given the circumstances of the sustained transmission of EBOV in the current outbreak in West Africa, increases in virulence may be associated with prolonged and uncontrolled epidemics of EBOV.

## Background

Ebola virus (EBOV) causes severe haemorrhagic fever in humans and non-human primates. Due to the high mortality rate, potential transmission from person-to-person contact and the lack of approved vaccines or anti-viral therapies, EBOV is classified as a hazard group 4 pathogen. The case fatality rate is related to the species of ebolavirus. EBOV has the highest case fatality rate (up to 90%) while Reston virus (RESTV) is not pathogenic for humans. However, RESTV can cause viral haemorrhagic fever in non-human primates and illustrates the potential zoonotic threat of ebolavirus [1–3]. Outbreaks occur sporadically, with the most recent outbreak occurring in Southern Guinea in 2014 [4], with fatality rates over 50%. This epidemic has now spread to Liberia, Sierra Leone and Nigeria [5], with one case reported in Senegal.

Ebola virus is an enveloped non-segmented negative-sense single-stranded RNA virus with a genome of 19 kb in length encoding several proteins including: nucleoprotein (NP), virion protein (VP) 35, VP40, the surface glycoprotein (GP_1,2_), VP30, VP24 and the RNA-dependent RNA polymerase (L). GP_1,2_ is the primary structural component and is exposed on the surface of the virus particle. The protein mediates host cell attachment and fusion [6]. A robust antibody response against GP_1,2_ is necessary for protection against lethal EBOV challenge [7]. The L protein is the catalytic subunit that forms an integral part of the polymerase complex that transcribes viral mRNA and replicates the viral genome. The combination and action of the Ebola virus gene products and their interactions with the host cell contribute to the severe haemorrhagic fever (see for example [6]).

There are several properties of the polymerase complex that contribute to virulence. The mRNA encoding GP_1,2_ is transcribed through two disjointed reading frames in the genome. These two reading frames are brought together by slippage of the viral polymerase complex at an editing site (a run of seven A residues) to insert an eighth A residue, generating an mRNA transcript that allows read-through translation of GP_1,2_ [8,9]. During EBOV infection and transcription of GP, approximately 20% of transcripts are edited, while the other 80% of unedited transcripts possess a premature stop codon. This results in the synthesis of a number of different products [10] including a truncated glycoprotein product (soluble GP - sGP), which is then secreted into the extracellular space. In a concept described as ‘antigenic subversion’ sGP has been proposed to induce a host antibody response that targets epitopes that sGP has in common with GP_1,2_, thereby allowing sGP to bind and compete for anti-GP_1,2_ antibodies [11]. The editing has been observed in both *in vitro* and *in vivo* models of infection [12].

Similar to other viruses with RNA genomes and which code an RNA dependent RNA polymerase, the L protein is unlikely to have extensive error correction, if at all. The molecular evolutionary rate for EBOV has been estimated at being between 2.2 × 10^-4^ and 7.06 × 10^-4^ nucleotide substitutions/site/year [13], whereas for nuclear genomes with error correction, it is approximately 10^-9^ nucleotide substitutions/site/year [14]. Therefore, the selection of phenotypic and hence genotypic variants of Ebola virus that can occupy new niches is facilitated through the high nucleotide substitution rate.

EBOV is proposed to be a zoonotic infection. Fruit bats are believed to be the natural reservoir and are not thought to develop disease [15]. A concern is that with increasing numbers of humans being infected in the current and potential future outbreaks, EBOV variants could be selected which are better adapted to be spread through human-to-human transmission.

## Results and discussion

In order to investigate how the EBOV genome changes with increasing pathogenicity, a forced evolution model was used in which EBOV was sequentially passaged *in vivo* using a guinea pig model of infection. EBOV is initially non-pathogenic in guinea pigs, but becomes more virulent and adapted to replicating in this host [16,17].

### Adaptation of EBOV to guinea pigs

**Figure 1:**
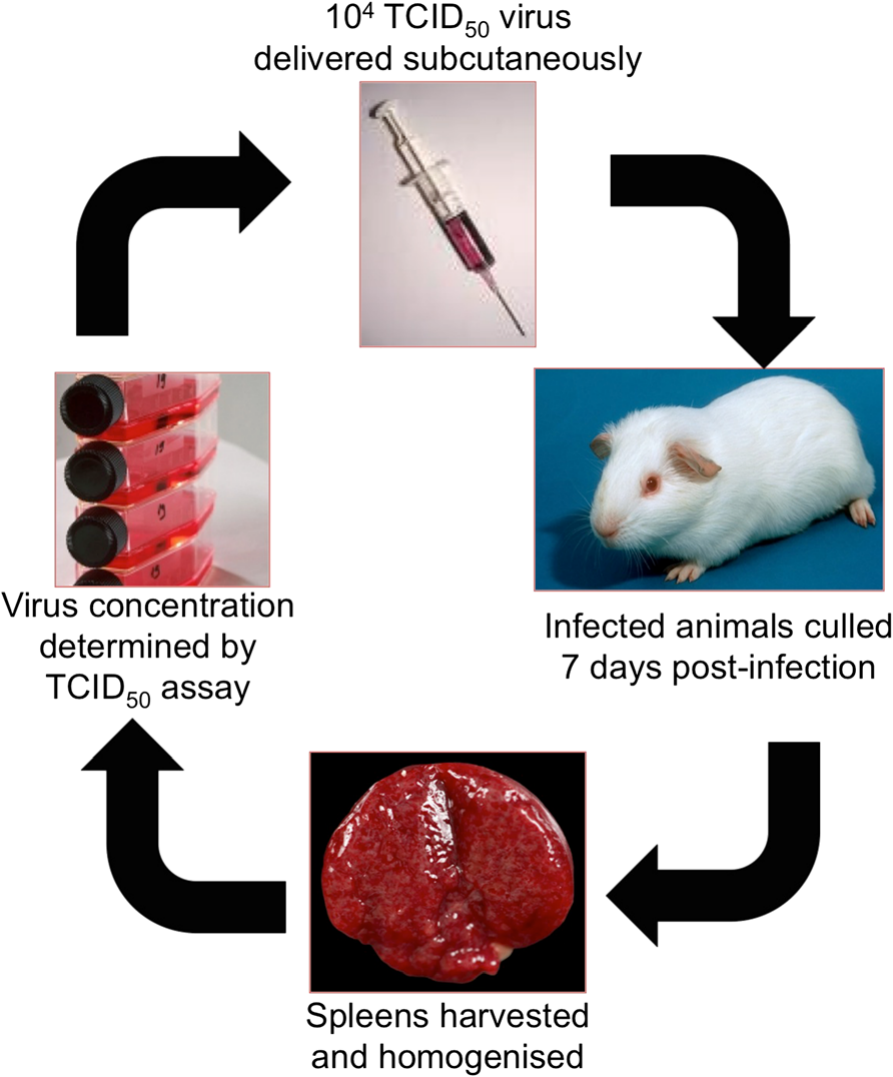
**Passaging of virus** ***in vivo*** **. In order to give a reproducible model of infection EBOV was passaged five times in guinea pigs in a forced evolution model.** There were 10 animals per group, where four animals were used for harvesting spleens for virus preparation and the remaining six animals used to measure clinical parameters.

**Figure 2:**
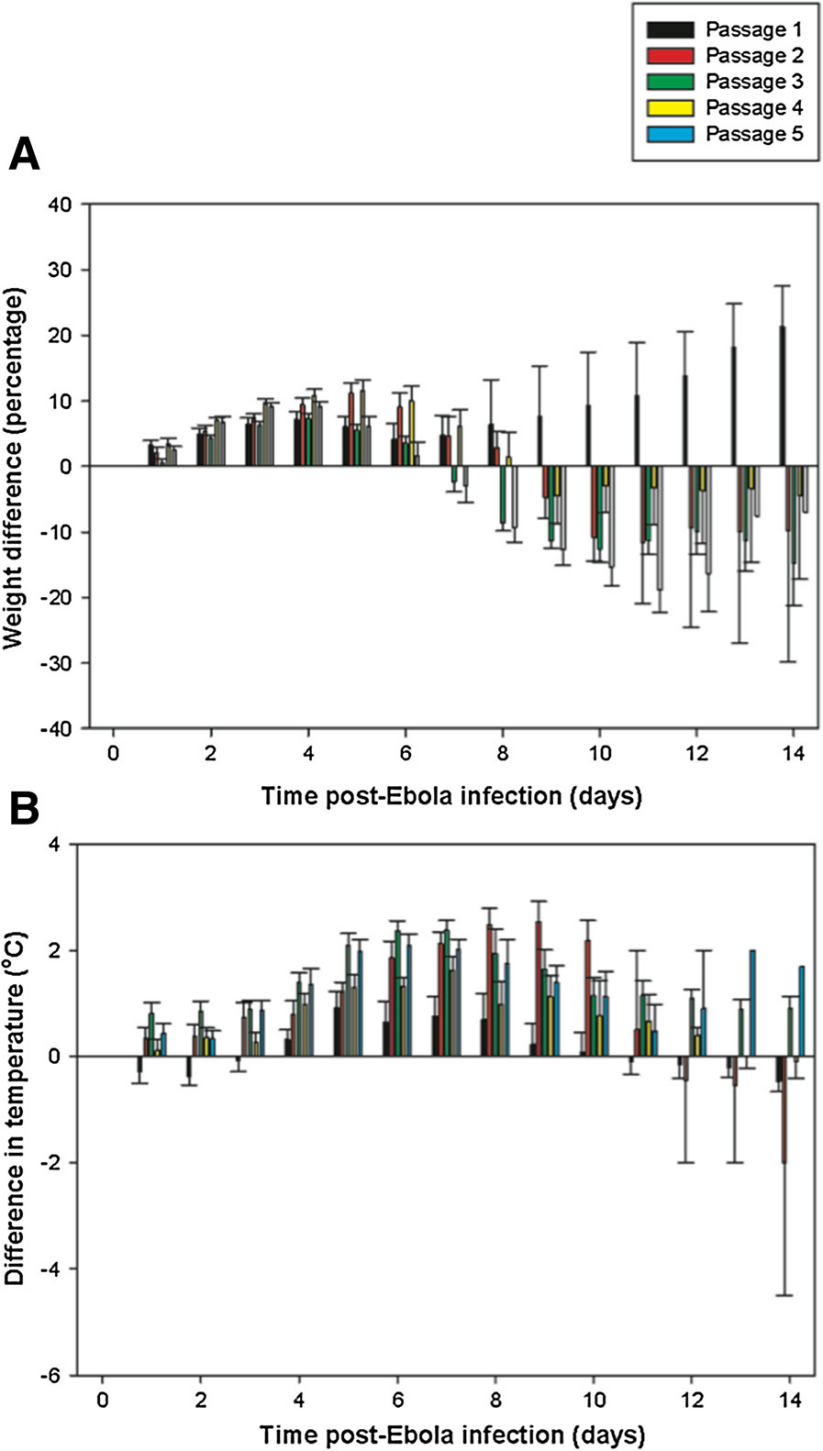
**Clinical data in the form of weight gain/loss and departure difference from EBOV-infected guinea pigs using virus that had been passaged from spleens harvested 7 days post infection: (A) weight and (B) temperature changes compared to day of challenge, compared to control uninfected animals.** Data points represent mean values from 10 animals up to day 7, and six animals up to day 14, with error bars denoting standard error.

**Figure 3:**
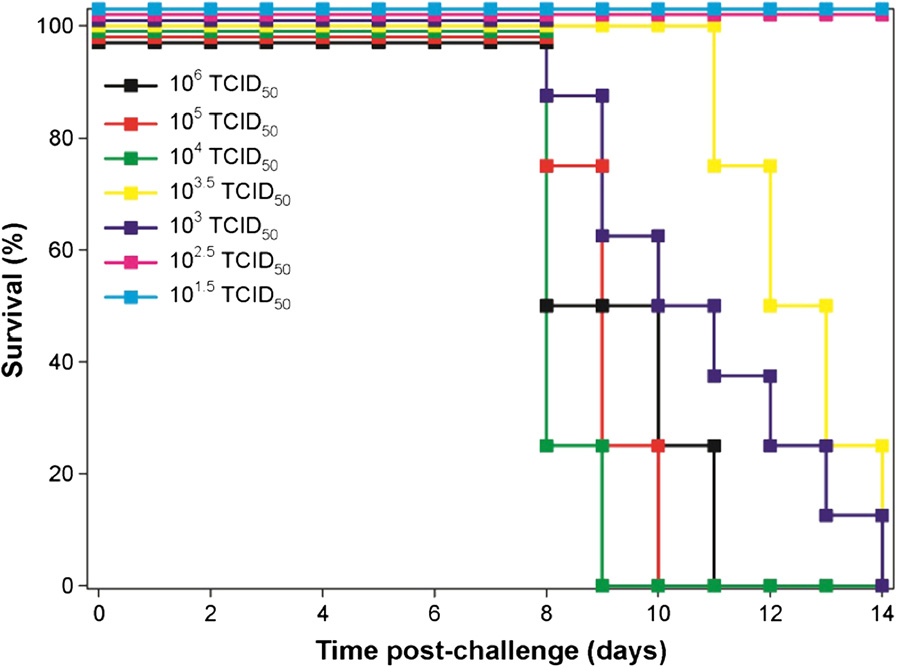
**Kaplan-Meier survival plot of EBOV-infected guinea pigs when different virus concentrations were used for challenge.** Survival studies lasted for 14 days.

Guinea pigs were infected with EBOV (ME718 strain) and the virus was serially passaged to develop uniform lethality in guinea pigs (Figure 1). There were 10 guinea pigs per passage. Four animals were used for the preparation of spleen homogenate for subsequent virus infection (culled 7 days post challenge) and six were taken forward for measuring survival rates and clinical parameters (for up to 14 days post challenge). Adaptation of EBOV to growth in the guinea pigs was achieved with serial passage involving a subcutaneous injection of 10^4^ TCID_50_ EBOV, with spleens harvested 7 days post infection (as a source of progeny virus). Virus titre was determined and a new inoculum prepared before administering 10^4^ TCID_50_ EBOV to a new group of guinea pigs. This was repeated until there was clinical and virological evidence that the virus adapted to the guinea pig host. Animals were observed for 2 weeks post infection. Weight data indicated that guinea pigs showed a minimal response to the initial challenge, whereas with subsequent passages weight loss exceeding 10% was observed (Figure 2A). Similarly, with temperatures the same responses were observed, where only after initial passage in the guinea pigs were temperature increases of between 1°C and 2.5°C observed (Figure 2B). At passage two several animals that met humane clinical endpoints displayed symptoms of hypothermia prior to being euthanized. Hypothermia has been previously observed in Rhesus macaques experimentally infected with EBOV via the aerosol route [18]. Six animals from each passage study that were scheduled to last 14 days post infection were used to assess mortality. By five passages, 75% mortality was observed with a challenge dose of 10^4^ TCID_50_. There was also no increase in viral titre in the spleen collected from animals culled at day 7 (Table 1) compared with the previous passage, indicating that the viral burden had peaked. The minimum lethal dose of the passaged virus was determined to be 10^3^ TCID_50_ (Figure 3).

**Table 1:** **The titre of EBOV in the spleens isolated from four guinea pigs taken from each passage increased, and then reached a plateau indicating that the virus had become adapted to grow in the guinea pig model**

**Passage number**	**Virus titre from spleen preparation (TCID**_**50**_ **)**	**Total PF reads**	**Reads mapping to the EBOV genome**	**Reads mapping to the Ebola genome (%)**	**Mortality (number of deaths/number challenged) (%)**
1	2.1 × 10^4^/spleen	2,429,959	4,298	0.18	0% (0/4)
2	3.0 × 10^7^/spleen	3,505,156	5,655	0.16	50% (2/4)
3	5.8 × 10^7^/spleen	3,453,615	9,736	0.28	0% (0/4)
4	6.1 × 10^7^/spleen	2,696,262	13,783	0.51	25% (1/4)
5	6.1 × 10^7^/spleen	3,264,859	12,060	0.37	75% (3/4)

The material prepared from the spleens was combined for subsequent infection of the next passage group. Note that the mortality associated with passage two is likely to be associated with the amount of spleen material used for inoculation (see Results and Discussion).

This method of adapting EBOV has been used by others and mortality was first shown to occur during passages three to four [19–21]. Complete lethality was then detected soon after, but ranged from passage four to seven [16,17,20,21]. While 50% lethality was seen in the second passage in the current study, this was most likely due to the low titres in the passage one material requiring a higher concentration of spleen homogenate to be delivered to the guinea pigs in order to achieve challenge with 10^4^ TCID_50_. This amount of material would have had adverse impacts due to lipid peroxidation, and protein oxidation and pro-apoptotic factors through cellular damage during preparation of the homogenate.

### Analysis of EBOV genome sequence with passage

Viral RNA was purified from spleens isolated from four guinea pigs from passage one through to five using a Qiagen Viral isolation kit. This allowed the safe transfer of nucleic acid from CL4 to CL2 and CL1 for further analysis. RNA was pooled from each passage and sequenced using MiSeq to avoid potential problems (associated with HiSeq) with the polyA carrier in the viral isolation kit. Sequence analysis indicated an increased proportion of sequence reads mapping to the EBOV genome with passage (Table 1). By our low stringency mapping approach, there were 4,298 reads in RNA sequenced from passage one material compared to 12,060 reads in RNA sequenced from passage five material. The decrease in percentage reads mapping to the Ebola genome in passage five compared to passage four may in part be due to the greater proportion of total reads mapped in passage five compared to passage four. Alternatively, these represent pooled samples and there was likely to be variation among individual clinical samples. Similarly, using a high stringency analysis of the alignments (see methods) we found 478 reads and 7,142 reads at passages one and five, respectively. This correlated well with the increase in viral titres observed with each passage. Nevertheless we regard viral titre as the definitive measure of viral load.

### Increased editing in the GP gene with passage

Sequence analysis identified editing in the mRNA encoding GP_1,2_, suggesting that viral mRNA might be co-purified or that there is editing of the genome itself during viral replication. Previous work has shown that approximately 20% of GP mRNA analysed from Ebola virus infected cells can be edited [8]. When we used TopHat [22] to align the sequencing reads to the viral genome and then searched the aligned reads for evidence of insertions within sequence reads mapping to the appropriate area on the viral genome. Analysis of the sequence data from each of the passages revealed that at passage one there were no insertions (0/23 sequence reads mapped to that region), at passage two approximately 15% of sequence reads had insertions (3/22 reads), similarly at passage three approximately 15% of reads had insertions (10/68 reads) but by passage four there was an increase to approximately 30% (36/124) and by passage five there were insertions in approximately 25% of the sequence reads mapping to that region (25/99 reads). Although the numbers of sequence reads were low, the data suggested that the proportion of full length mRNA encoding GP_1,2_ increased with sequential passage and this may be associated with the gain of virulence observed with sequential passage in the guinea pig model. One interpretation of this data is that the amount of GP_1,2_ was limiting in early passages. As the proportion of edited mRNA increased so more GP_1,2_ was available for virus assembly and on virus particles and this contributed to the increase in progeny virus observed in the later passages. However, a more stringent analysis of sequence reads that mapped to the relevant region of the glycoprotein gene showed additional A residues at the following rates: 0 out of 2 mapped reads for passage one, 2 out of 3 (66%) mapped reads at passage two, 17 out of 45 (37.8%) reads at passage three, 45 out of 115 (39.1%) mapped reads for passage four and 37 out of 91 (40.7%) mapped reads for passage 5. Thus, an analysis of those reads mapped at a higher stringency indicated an increased rate of editing both overall and increasing with passage. However, the low number of reads overall means these observations must be treated with caution.

### Nucleotide substitutions become established with passage

EBOV sequence at each passage was analysed for coverage and variants using QuasiRecom [23], allowing us to determine consensus nucleotide at each position, map the frequency of minor variants. Thus we determine a consensus sequence for the virus at each passage and compare these consensus sequences to the published EBOV sequence at each particular passage. Examining the minor variants allowed us to determine if changes became established. For example, a minor variant at passage three that is not present at passage four indicates the sequence change did not become established. Thus we could distinguish whether a coding change became established with passage or not. There were two major types of substitutions; those that appeared in passages two to four and were selected against by passage five (Table 2); or alternatively substitutions that became established by passage five (Table 3). Some of these correlated with previous point mutational analysis, thus placing confidence in the approach.

**Table 2:** **Amino acid substitutions that are the predominant change in the virus population analysed during an individual passage**

**NP**									
	**191**	**323**	**414**	**566**					
**EBOV**	**W**	**V**	**L**	**N**					
P1	R	D	R	N					
P2	?	V	L	S					
P3	W	V	L	S					
P4	W	V	L	S					
P5	W	V	L	S					
**VP35**									
	**129**	**204**	**246**						
**EBOV**	**S**	**N**	**I**						
P1	P	D	A						
P2	S	D	I						
P3	S	D	I						
P4	S	D	I						
P5	S	D	I						
**VP40**									
	**15**	**66**	**259**						
**EBOV**	**E**	**P**	**M**						
P1	Q	S	M						
P2	?	P	R						
P3	E	P	M						
P4	E	P	M						
P5	E	P	M						
	**sGP**								
**sGP/GP**	**GP**								
	**1**	**11**	**49**	**92**	**187**	**203**	**465**	**493**	**638**
**EBOV**	**M**	**R**	**D**	**V**	**P**	**V**	**I**	**S**	**R**
P1	?	?	?	L	P	?	T	S	K
P2	K	?	?	V	L	I	I	P	R
P3	M	H	N	V	P	I	I	S	R
P4	M	R	N	V	P	I	I	S	R
P5	M	R	N	V	P	I	I	S	R
**sGP/GP**	**GP**								
	**652**	**668**							
**EBOV**	**Y**	**Y**							
P1	Y	Y							
P2	F	C							
P3	Y	Y							
P4	Y	Y							
P5	Y	Y							
**VP30**									
	**214**	**248**							
**EBOV**	**L**	**Q**							
P1	P	?							
P2	L	R							
P3	L	Q							
P4	L	Q							
P5	L	Q							
**VP24**									
	**26**	**29**	**43**	**218**					
**EBOV**	**L**	**F**	**A**	**K**					
P1	F	V	P	R					
P2	F	F	A	?					
P3	F	F	A	K					
P4	F	F	A	K					
P5	F	F	A	K					
**L**									
	**30**	**38**	**161**	**525**	**537**	**538**	**669**	**705**	**707**
**EBOV**	**G**	**N**	**R**	**N**	**K**	**L**	**I**	**M**	**G**
P1	?	?	W	N	R	P	?	?	?
P2	G	N	R	N	K	L	S	?	?
P3	W	N	R	D	K	L	I	T	?
P4	?	?	R	N	K	L	I	M	G
P5	G	K	R	N	K	L	I	M	A
**L**									
	**826**	**868**	**879**	**930**	**940**	**943**	**993**	**1096**	**1271**
**EBOV**	**S**	**S**	**F**	**T**	**L**	**I**	**T**	**L**	**Y**
P1	Y	S	?	?	?	?	?	?	?
P2	S	S	F	T	L	I	A	S	Y
P3	S	S	F	T	L	I	T	L	Y
P4	S	P	L	T	L	R	T	L	Y
P5	S	S	F	A	P	I	T	L	STOP
**L**									
	**1308**	**1478**	**1546**	**1733**	**1763**	**1949**	**1998**	**2144**	**2151**
**EBOV**	**S**	**N**	**A**	**F**	**L**	**H**	**S**	**N**	**F**
P1	?	?	?	?	?	?	?	N	-
P2	P	I	A	F	L	Q	S	N	V
P3	S	N	A	F	L	H	S	N	V
P4	S	N	A	Y	P	?	?	N	V
P5	?	H	E	F	L	H	T	K	V
**L**									
	**2197**								
**EBOV**	**L**								
P1	-								
P2	L								
P3	L								
P4	P								
P5	L								

The protein name is indicated, as is the amino acid position. EBOV is the amino acid present in the input sequence.

**Table 3:** **Amino acid substitutions that are the predominant change in the virus population analysed at passage 5**

**NP**										
	**566**									
**EBOV**	**N**									
P1	N									
P2	S									
P3	S									
P4	S									
P5	S									
**VP35**										
	**204**									
**EBOV**	**N**									
P1	D									
P2	D									
P3	D									
P4	D									
P5	D									
**No amino acid mutations for VP40**										
**sGP**										
	**49**	**203**								
**EBOV**	**D**	**V**								
P1	?	?								
P2	?	I								
P3	N	I								
P4	N	I								
P5	N	I								
**No amino acid mutations for VP30**										
**VP24**										
	**26**									
**EBOV**	**L**									
P1	F									
P2	F									
P3	F									
P4	F									
P5	F									
**L**										
	**38**	**707**	**930**	**940**	**1271**	**1478**	**1546**	**1998**	**2144**	**2151**
**EBOV**	**N**	**G**	**T**	**L**	**Y**	**N**	**A**	**S**	**N**	**F**
P1	?	?	?	?	?	?	?	?	N	-
P2	N	?	T	L	Y	N	A	S	N	V
P3	N	?	T	L	Y	N	A	S	N	V
P4	N	G	T	L	Y	N	A	?	N	V
P5	K	A	A	P	STOP	H	E	T	K	V
**L**										
	**2186**									
**EBOV**	**M**									
P1	-									
P2	M									
P3	M									
P4	M									
P5	T									

The protein name is indicated, as is the amino acid position. EBOV is the amino acid present in the input sequence.

Some viral proteins accumulated substitutions whereas other did not. No substitutions were observed in either VP40 or VP30 by passage five. VP40 is a viral matrix protein with multiple roles in the virus life cycle, associating with both cellular and other viral proteins including the ribonucleoprotein complex. It is also involved in virus assembly and release (for example, [24,25]), and thus may be evolutionary constrained. Likewise VP30 is a transcription factor and modulates interaction with NP and VP35 (for example, [26]) and may operate independently of the host cell for function.

Some substitutions were present in early passages but were lost by passage five. For example in VP35 at passages one and two, the predominant amino acid at position 84 was a Gly rather than the Glu found in the input sequence. By passages three, four and five this was again a Glu. In VP40 at passages two and three the predominant amino acid at position 16 was a Pro rather than the Ala found at passages one and two and the input sequence. By passages four and five, this had become an Ala again. Some of these changes have been previously associated with virulence. For example, in VP24, the predominant amino acid at position 163 changes from a Lys to Arg in passage three (and then Lys becomes dominant again in passages four and five), which is a conserved substitution. This substitution was described previously by Kugelman et al., who postulated that this amino acid change in VP24 might modulate interaction with other proteins instead of having an effect on structural stability [27].

A number of amino acid substitutions became established during adaptation and were present in passage five (Table 3). For example, the predominant amino acid at position 26 in VP24 becomes a Phe in place of a Leu. This substitution has previously been identified as being responsible for an increase in virulence in the guinea pig model using reverse genetics [28], and thus places confidence in the analysis of consensus sequence to detect biologically relevant variations. Using data from the stringent analysis, the frequency of amino acid substitution (amino acid changes/number of amino acid in the ORF) appeared to be of the same order of magnitude for each of the proteins that had an amino acid substitution(s) by passage five (Table 3). For NP this was 0.001, VP35 was 0.003, sGP was 0.006, VP24 was 0.004 and L was 0.005. However, there were no coding mutations in VP40 and VP30. While we observed 11 coding changes by passage five in the L protein, a study investigating adaptions of EBOV to a mouse model highlighted two coding changes and one silent change. This may reflect a difference in adaption of the virus to the two hosts.

As noted by Ebihara et al., additional mutations in the L protein are likely to contribute to virulence by affecting viral RNA synthesis [29]. These may also mediate both viral and host cell interactions. A similar situation has been described for influenza A virus where a cellular protein is critical for polymerase activity and transmission between different species and interaction of this protein with the polymerase is determined by a single amino acid substitution [30,31]. However, with that said, data indicates that VP24 associates with a number of different cellular proteins that may be critical for its function inside virus infected cells (for example, [32–35]). It is interesting to speculate that the adaptation of the L protein to the new host may be correlated with the increase in RNA editing activity to transcribe the GP_1,2_ mRNA. This editing activity of the polymerase complex maybe associated with and mediated by a host cell factor, although both cis-acting factors on the EBOV genome and VP30 have been implicated in this process [36].

In this study, a virus (EBOV) that was not initially virulent in guinea pigs was serially passaged and became more pathogenic in its new host. Several alterations in the amino acid coding sequence were associated with this increase in virulence. A reverse genetics approach would precisely characterise these changes and their linkage with pathogenicity and virulence. Interestingly, this approach was used in a mouse model study of EBOV to investigate the molecular determinants of virulence [29]. There are several implications of our research for the biology of EBOV and associated outbreaks. EBOV causes a zoonotic infection [1,37] and humans have been considered a dead end host, with the long-term survival of EBOV in nature likely being dependent on its ability to persist in its natural host. Our data suggest that the selection pressures at the initial stages of replication in a new host are different from those when the virus becomes established, and this may be dependent on population size, density and route of transmission. Therefore, as EBOV is so pathogenic in humans, one possibility for a sustained human-to-human transmission scenario might be selection of variants that are less pathogenic and that could lead to a more long-term infection of the population, thus allowing EBOV to persist. However, balanced with this are the social aspects of infection. For example, where humans tend to gather to grieve, suggesting that a reduction in pathogenicity is not the only way the virus can be marinated long term in the human population. Our study can be considered a model for the initial jump of EBOV from a reservoir into a new host, where selection pressure may be at its highest. The data presented in this study, using a forced evolutionary and transmission model, would suggest that the initial evolutionary trajectory of EBOV in a new host leads to a gain in virulence. Given the circumstances of the sustained transmission of EBOV in the current outbreak in West Africa, increases in virulence maybe associated with prolonged and uncontrolled epidemics of EBOV.

## Conclusions

Ebolaviruses are zoonotic, and when certain species such as EBOV infect humans, they can have high case fatality rates. The implication of this is that transmission of the virus is contained when humans live in isolated villages. However, in the advent of large-scale outbreaks in higher population densities there is a greater chance of the virus becoming more adapted for growth and transmission in the human population. Transmission studies with influenza virus using animal models have identified key viral mutations involved in this process and have illustrated how these may be used as predictors during an influenza infection/pandemic [38–40]. Such approaches can be applied to EBOV infection. Here we used a guinea pig model to show how RNAseq and consensus assembly of genomes can be used to identify genome modification and coding changes that are associated with increasing virulence, pathogenesis and disease pathology. Such approaches may indicate biomarkers that can be monitored during outbreaks in order to evaluate the risk of adaptation.

## Methods

### Animals

Female Dunkin-Hartley guinea pigs were used for animal infection studies, with weights of 250 g to 350 g (Harlan Laboratories, UK). Before procedures involving the manipulation of animals, guinea pigs were anesthetised with 1.5% to 2% isofluorane in an induction change until full sedation was achieved. Animals infected with EBOV were housed within an isolator under climate-control conditions in an animal containment level 4 (CL4) room. Food and sterile water were available *ad libitum*. All procedures were undertaken according to the United Kingdom Animals (Scientific Procedures) Act 1986. A power calculation along with Fisher’s exact test were performed using software G*Power ver.3.0.10 to determine group sizes for the experiments. A minimum group size of six met a power of 0.8 and alpha at 0.05. We also note that from previously published work in this area, that all animals become infected with EBOV at later passages. There were 10 guinea pigs depending on the group for each passage of the virus and a control group. From a practical standpoint of working at CL4 this number also represented the maximum number of animals that could be processed at the time. Of these animals, four were killed at day 7 post infection for preparation of virus and six to eight were carried on and used to measure clinical parameters. The study was performed under a UK Home Office Project License conforming to the Animal Procedures Act. Ethical review was performed by the Public Health England Animal Welfare and Ethical Review Board.

### Virus

The EBOV Zaire ME718 strain was used in this work. This was originally isolated during an outbreak in October 1976 [41] in Yambuku, Mongala Province in what is currently the northern Democratic Republic of the Congo, and it was simultaneously reported in three publications [42–44]. Virus stocks used for this work were grown in VeroE6 cells (European Collection of Cell Cultures, UK) cultured in Leibovitz’s L15 (L15) media containing 2% fetal calf serum (FCS), and aliquots were stored at -80°C. Virus titres were determined by 100-fold dilution with L15 media without any FCS added. A total of 100 μL of each dilution was overlaid onto semi-confluent cell monolayers in four replicate 12.5 cm^2^ tissue culture flasks and left to absorb for 1 h. A volume of 5 mL media was then added and cells were incubated at 37°C for 7 to 8 days. Cytopathic effects were determined by microscopy, and the results from each dilution were used to calculated 50% tissue culture infective dose (TCID_50_) using the Reed-Muench method [45].

### Animal challenge

EBOV stock was diluted in sterile PBS to prepare the relevant dose of virus in a 0.2 mL volume. For passaging experiments (required for virus adaptation), the dose delivered was 10^4^ TCID_50_. Surplus inoculation was made to confirm concentration via back titration in cell culture. Guinea pigs were sedated, and subcutaneously inoculated with the virus suspension in the lower right quadrant of the back, then returned to their cages and monitored for adverse effects caused by the injection of the anaesthetic until the animals fully recovered. Negative control groups were injected with the same volume of PBS.

### Observations and monitoring

Animals were monitored at least twice daily, and observations (swelling at injection site, movement, breathing, food intake, water intake and appearance) recorded for the duration of the study. A set of humane clinical end points were defined (20% weight loss, or 10% weight loss and a clinical symptom) which indicated that the animal would be euthanised to prevent any unnecessary suffering. Weights of the animals were taken daily, and temperatures recorded using a pre-inserted temperature chip.

### Necropsy and tissue collection

Animals were humanely euthanised by intraperitoneal injection of 200 mg/mL pentobarbital sodium. Necropsies were performed within a flexible-film isolator in the animal CL4 facility. Spleens were removed from four out of the 10 guinea pigs at each passage and stored at -80°C. For processing, samples were transported to the *in vitro* CL4 laboratory and thawed at room temperature. Spleens were homogenised by vigorously passing through a 500 mm cell strainer (Corning, UK). The resultant suspension was clarified by centrifugation at 400 g for 10 min to remove cell debris. The supernatant was collected, aliquoted and stored at -80°C. One vial was used to assess virus concentration by TCID_50_ assay.

### RNA preparation

Spleen homogenate from each passage was added to AVL buffer (Qiagen, UK) and removed from the CL4 laboratory for processing. RNA was isolated using a viral RNA extraction kit following the manufacturer’s instructions (Qiagen, UK). Confirmation of EBOV-specific RNA extraction was achieved by use of a block-based PCR assay. The RNA was then pooled from individual guinea pigs for each passage for sequencing analysis.

### Sequencing and alignment

Extracted RNA was DNase treated with Turbo DNase (Ambion) using a rigorous protocol. RNA–Seq libraries were prepared from the resultant RNA using the Epicentre ScriptSeq v2 RNA-Seq Library Preparation Kit. RNA (50 ng) was used as input and following 12 cycles of amplification, libraries were purified using AMPure XP beads. Each library was quantified using Qubit and the size distribution assessed using the Agilent 2100 Bioanalyser. These final libraries were pooled in equimolar amounts using the Qubit and Bioanalyzer data. The quantity and quality of the pool was assessed by Bioanalyzer and subsequently by qPCR using the Illumina Library Quantification Kit from Kapa on a Roche Light Cycler LC480II according to manufacturer’s instructions. The pool of libraries was sequenced on one flowcell of the MiSeq at 2 × 150 based paired-end sequencing with v2 chemistry. Due to the high-expected levels of polyA RNA introduced during the RNA extraction 15% of phiX library was loaded onto the flowcell for sequencing to produce a more balance base composition. The Fastq files were QC filtered as follows. Initial processing and quality assessment of the sequence data were performed using an in-house pipeline (developed by Richard Gregory). Briefly, base calling and de-multiplexing of indexed reads was performed by CASAVA version 1.8.2 (Illumina). The raw fastq files were trimmed to remove Illumina adapter sequences using Cutadapt version 1.2.1 [46]. The option ‘-O 3’ was set, so the 3’ end of any reads which matched the adapter sequence over at least 3 bp was trimmed off. The reads were further trimmed to remove low quality bases, using Sickle version 1.200 with a minimum window quality score of 20. After trimming, reads shorter than 10 bp were removed. If both reads from a pair passed this filter, each was included in the R1 (forward reads) or R2 (reverse reads) file. If only one of a read pair passed this filter, it was included in the R0 (unpaired reads) file. The data post QC analysis was uploaded to the European Nucleotide Archive with study accession number PRJEB7406, with a direct URL link of [47]. There was significant variation in the amount of viral RNA present in each sample. This presented challenges when obtaining a consensus alignment for the viral genetic material. Therefore, the sequence data were aligned using several approaches before settling on two distinct types of analysis, a low stringency and high stringency approach. Initially we used a low specificity approach in order to gain maximum data return while accepting that some of the alignment output may be of low quality. In this approach, the sequence reads were simply aligned to the viral genome using TopHat [22]. In a second, highly stringent approach, we used Bowtie2 to map the reads to the viral genome. We then parsed the output to discard reads that did not map in a proper mate pair before removing PCR duplicates - both steps were done using Sequence Alignment Map (SAM) tools. At this stage any reads less than 25 nucleotides were not considered. In either case, the output files were converted into SAM files to allow manual searching for sequence reads corresponding to the region of the G glycoprotein known to contain non-templated insertions. For this we used the UNIX command ‘grep’ to search within the SAM file for exact matches to the sequence ‘CTAAAAAAACC’ (that is, unedited sequence) and for exact matches to the sequence ‘CTAAAAAAAACC’ (that is, edited sequence). In addition, the reads aligned to the virus genome were analysed using QuasiRecomb [23]. We used the command line instruction ‘java -XX:NewRatio = 9 -Xms4G -Xmx26G -jar QuasiRecomb.jar -i ebola_passage1.bam –coverage’. This generated a coverage file indicating how many sequence reads covered each nucleotide of the genome and the frequency of each of the four nucleotides at each position on the virus genome. From this output we were able to generate a consensus sequence for the virus at each passage, which was later analysed using CLUSTAL [48].
